# Substance P-expressing excitatory interneurons in the mouse superficial dorsal horn provide a propriospinal input to the lateral spinal nucleus

**DOI:** 10.1007/s00429-018-1629-x

**Published:** 2018-03-01

**Authors:** Maria Gutierrez-Mecinas, Erika Polgár, Andrew M. Bell, Marine Herau, Andrew J. Todd

**Affiliations:** Institute of Neuroscience and Psychology, College of Medical, Veterinary and Life Sciences, University of Glasgow, Glasgow, G12 8QQ UK

**Keywords:** Spinal cord, Pain, Lamina II, Intraspinal injection, Tac1, AAV

## Abstract

The superficial dorsal horn (laminae I and II) of the spinal cord contains numerous excitatory and inhibitory interneurons, and recent studies have shown that each of these groups can be divided into several neurochemically distinct populations. Although it has long been known that some neurons in this region have intersegmental (propriospinal) axonal projections, there have been conflicting reports concerning the number of propriospinal cells and the extent of their axons. In addition, little is known about the neurochemical phenotype of propriospinal neurons or about the termination pattern of their axons. In the present study we show, using retrograde tracing, that around a third of lamina I–II neurons in the lumbar enlargement project at least five segments cranially. Substance P-expressing excitatory neurons are over-represented among these cells, accounting for one-third of the propriospinal neurons. In contrast, inhibitory interneurons and excitatory PKCγ neurons are both under-represented among the retrogradely labelled cells. By combining viral vector-mediated Cre-dependent anterograde tracing with immunocytochemistry, we provide evidence that the lateral spinal nucleus (LSN), rather than the superficial dorsal horn, is the main target for axons belonging to propriospinal substance P-expressing neurons. These findings help to resolve the discrepancies between earlier studies and have implications for the role of the LSN in pain mechanisms.

## Introduction

The spinal dorsal horn receives a highly ordered input from primary afferents coding for various somatosensory modalities. This information is processed by local interneurons and modulated by descending axons before being transmitted to the brain, and to reflex circuits in deeper laminae. The superficial dorsal horn (laminae I–II) has attracted particular interest, because it is the main target for nociceptive afferents (Peirs and Seal 2016). This region contains densely packed neurons, the vast majority of which have axons that remain in the spinal cord, and are, therefore, defined as interneurons (Todd 2017). Interneurons in laminae I–II can be divided into two broad classes: inhibitory (GABAergic/glycinergic) cells account for ~ 25% of the total population, while excitatory (glutamatergic) cells make up the remaining ~ 75% (Polgár et al. 2013). Recent studies have demonstrated that each of these classes can be subdivided into neurochemically distinct populations, and have begun to attribute specific functional roles to these populations (Todd 2017).

Although all interneurons in laminae I–II appear to have axons that arborise locally (Grudt and Perl 2002; Yasaka et al. 2010), it has been reported that these cells can also have propriospinal axons that extend for several segments. However, while there have been several studies of propriospinal neurons in the deep dorsal horn (laminae III–VI) and ventral horn (Flynn et al. 2011; Brockett et al. 2013), much less is known about the organisation of intersegmental connections in the superficial laminae. Bice and Beal (1997b) reported that many lamina I and II neurons in the L1 segment of the rat were retrogradely labelled following application of Fluorogold to the hemisected spinal cord at T5. When neurons with supraspinal projections were accounted for (Bice and Beal 1997a), their findings suggested that around 10% of neurons in laminae I–II have long propriospinal axons, but do not project to the brain. However, Petko and Antal (2000, 2012), who made restricted injections of tracers into different regions of the rat L4 dorsal horn, concluded that neurons in laminae I–II had relatively short axonal projections to the dorsal horn of other segments, with very few extending more than a segment rostrally or caudally. One possible explanation for the discrepancy between these findings is that neurons in laminae I–II have long propriospinal axons that terminate outside the dorsal horn, for example, in the lateral spinal nucleus (LSN), which is known to be innervated by dorsal horn neurons (Cliffer et al. 1988).

The first aim of the present study was to determine whether the mouse has a similar propriospinal projection pattern involving superficial dorsal horn neurons to that described for the rat (Bice and Beal 1997b), and if so, whether this involves excitatory and/or inhibitory neurons. Substance P-containing axons originating from dorsal horn neurons are thought to innervate the LSN (Cliffer et al. 1988; Giesler and Elde 1985), and we have recently shown that substance P is expressed by a distinct subset of the excitatory interneurons in laminae I–II (Gutierrez-Mecinas et al. 2017). We, therefore, looked for evidence that these cells were included among those with long propriospinal projections, and we compared these to a different population of excitatory interneurons, those that express protein kinase Cγ (PKCγ). To identify substance P-expressing neurons, we used mice with Cre recombinase knocked into the Tac1 locus, which codes for preprotachykinin A (PPTA, the precursor for substance P). We performed intraspinal injection of adenoassociated viruses (AAVs) with Cre-dependent expression cassettes, because we have shown that this strategy reveals dorsal horn neurons that express PPTA in adult animals (Gutierrez-Mecinas et al. 2017). This approach also allowed us to follow axons belonging to these cells, and, therefore, to identify the targets of the propriospinal substance P neurons.

## Materials and methods

### Animals

All experiments were approved by the Ethical Review Process Applications Panel of the University of Glasgow, and were performed in accordance with the European Community directive 86/609/EC and the UK Animals (Scientific Procedures) Act 1986.

To identify propriospinal neurons with axons that ascended for at least five segments, and to determine whether these included substance P-expressing cells, we performed intraspinal injections of the retrograde tracer cholera toxin B subunit (CTb) and of an AAV that codes for a Cre-dependent form of eGFP (AAV.flex.eGFP) in Tac1^Cre^ knock-in mice (Harris et al. 2014). The virus (AAV serotype 1) encodes an inverted sequence for eGFP between pairs of heterotypic LoxP sites with anti-parallel orientation (Atasoy et al. 2008) and is under transcriptional control by the CAG promoter. In infected cells that express Cre at the time of injection, there will be permanent reversal of the coding sequence, resulting in expression of eGFP (Gutierrez-Mecinas et al. 2017). Four Tac1^Cre^ mice of either sex aged 6–9 weeks (16–27 g) were anaesthetised with isoflurane and placed in a stereotaxic frame. The vertebral column was exposed, and each mouse received two intraspinal injections through a glass micropipette (inner diameter of tip 40 µm) into the dorsal horn on the right side, 300–400 µm lateral to the midline and at a depth of 300 µm below the pial surface. The first injection consisted of 150 nl of 1% CTb, and was targeted on the T13/L1 spinal segments by inserting the pipette between the T11 and T12 vertebrae. The second injection consisted of 300 nl of AAV.flex.eGFP (17.2 or 8.6 × 10^8^ gene copies; Penn Vector Core, Philadelphia, PA, USA) and was targeted on the L5 spinal segment by injecting between T13 and L1 vertebrae. Both injections were administered at a rate of 30 nl/min. The wound was closed, and animals were allowed to recover with appropriate analgesia. After a survival period of 3–4 days, the mice were reanaesthetised with pentobarbitone (30 mg, intraperitoneally) and perfused through the heart with a fixative that contained 4% freshly depolymerised formaldehyde in phosphate buffer. A relatively short survival time was used in these experiments because of the possibility that levels of CTb in retrogradely labelled neurons could decline at longer post-operative times. The spinal cord segments from T12–L6 were removed and post-fixed for 2 h in the same fixative.

To investigate the axonal projections of substance P-expressing neurons, we made a single injection of AAV.flex.eGFP (8.6 or 4.3 × 10^8^ gene copies in 300 nl) into the L5 spinal cord segments of four additional male Tac1^Cre^ mice aged 6–8 weeks (23–28 g). The procedure was the same as that described above, except that these animals did not receive an injection of CTb. Following a survival time of 7 days (*n* = 2) or 29 days (*n* = 2), the mice were reanaesthetised and fixed by perfusion as described above. In these cases, the entire spinal cord and brain were removed and post-fixed in the same way. In addition, the L4 and L5 dorsal root ganglia on the right (ipsilateral) side were removed from the two mice that survived 29 days.

### Immunocytochemical processing and confocal microscopy

In the mice that received injections of both CTb and AAV.flex.eGFP, a block of tissue corresponding to the T13–L1 segments was cut into 60 µm-thick transverse sections with a vibrating blade microtome (Leica VT 1200). The sections were cut sequentially into 4 bottles, and the sections from one bottle from each mouse were incubated in anti-CTb at 1:200,000 and processed with an immunoperoxidase method to reveal the CTb injection site, as described previously (Cameron et al. 2015).

Another block of tissue corresponding to the L4–5 segments from each of these mice was cut transversely into four series of 60 µm-thick transverse sections, three of which were used in the present study. The sections were reacted for immunofluorescence staining as described previously (Gutierrez-Mecinas et al. 2017). Briefly, they were incubated for 3 days at 4 °C in mixtures of primary antibodies and then overnight in mixtures of species-specific secondary antibodies that were raised in donkey. The secondary antibodies were conjugated to Alexa 647, Rhodamine Red or Pacific Blue (Jackson ImmunoResearch, West Grove, PA) and were used at 1:500 (Alexa 647), 1:100 (Rhodamine Red) or 1:200 (Pacific Blue). After immunoreaction, the sections were mounted in antifade medium and stored at − 20 °C. All antibodies were diluted in phosphate-buffered saline that contained 0.3% Triton X-100 and 5% normal donkey serum. The sources and dilutions of the primary antibodies are shown in Table 1. The combinations of primary antibodies used in this part of the study were as follows: (1) CTb and NeuN (chicken antibody); (2) CTb, NeuN (guinea-pig antibody), and Pax2; and (3) CTb, NeuN (guinea-pig antibody), and PKCγ. The sections from the first reaction were subsequently counterstained with the nuclear stain 4′,6-diamidino-2-phenylindole (DAPI).

**Table 1:** Antibodies used in this study

Antibody	Species	Catalogue no	Dilution	Source
CTb	Goat	703	1:5000 1:200,000^a^	List biological
NeuN	Chicken	266 006	1:1000	Synaptic systems
NeuN	Guinea pig	266 004	1:500	Synaptic systems
Pax2	Rabbit	716 000	1:1000	Life technologies
PKCγ	Rabbit	sc211	1:1000	Santa cruz biotechnology
GFP	Chicken	Abcam	1:1000	ab13970
Substance P	Rat	OBT06435	1:200	Oxford biotech
Somatostatin	Rabbit	T-4103	1:1000	Peninsula
VGLUT2	Guinea pig	ab2251	1: 5000	Millipore
CGRP	Guinea pig	Bachem	1:10,000	T-5027

^a^For immunoperoxidase reaction

From the animals that received only injections of AAV.flex.eGFP, transverse sections were cut from each spinal segment from T13 to S2, as well as from some of the more rostral spinal segments. In addition, coronal sections of brain that included the medulla, lateral parabrachial area (LPb) and periaqueductal grey matter (PAG) were also obtained from these mice. Sections were reacted for immunofluorescence as described above. They were incubated in antibodies against eGFP and VGLUT2, which were used to confirm the presence of axonal boutons originating from eGFP^+^ neurons, most of which are excitatory (Gutierrez-Mecinas et al. 2017), and also to define the location of the LSN, which contains a high density of VGLUT2-immunoreactive terminals (Olave and Maxwell 2004; Todd et al. 2003). Immunostaining with the eGFP antibody was used to amplify the fluorescence of eGFP, to make axonal staining more clearly visible, and the eGFP antibody was detected with a secondary antibody conjugated to Alexa 488 (1:500). In some cases, antibodies against substance P and somatostatin were also used to identify axons that were likely to have originated from neurons in laminae I–II that express both these neuropeptides (Gutierrez-Mecinas et al. 2017). Some sections from the L4-5 segments were reacted with antibodies against calcitonin gene-related peptide (CGRP), to test whether eGFP-labelled axons included substance P-expressing primary afferents, which co-express CGRP (Ju et al. 1987). The ipsilateral L4 and L5 dorsal root ganglia from the mice that survived 29 days after AAV.flex.eGFP were mounted intact in antifade medium.

Tissue with fluorescent labelling was scanned with a Zeiss LSM710 confocal microscope equipped with Argon multi-line, 405 nm diode, 561 nm solid state, and 633 nm HeNe lasers.

### Neurochemical characterisation of neurons in L5 that were retrogradely labelled from T13/L1 segments

From the mice that received both CTb and AAV.flex.eGFP injections, two L4–5 sections from each animal that had been reacted with each antibody combination were selected for scanning and analysis, and this selection was made before CTb immunostaining was viewed. For the quantitative analysis of eGFP-expressing cells, we chose two sections that contained high numbers of eGFP^+^ cells, because some of the sections from the L4-5 block did not include the AAV.flex.eGFP injection site. Scans were obtained through a 40× oil-immersion lens (numerical aperture 1.3) to generate *z* series (1 µm *z* separation), such that the entire cross-sectional area of the ipsilateral dorsal horn through the full thickness of the section was included. The confocal scans were analysed with Neurolucida for Confocal (MBF Bioscience, Williston, VT, USA) using a modification (Gutierrez-Mecinas et al. 2017) of the disector method (Sterio 1984). The reference and look-up sections were set between 10 and 15 µm apart. Because all quantitative analyses were performed on laminae I–II, we initially plotted the outline of the dorsal horn grey matter, and then located the lamina II–III border, either by the relatively low density of neurons in the inner half of lamina II (IIi), or by the presence of a plexus of PKCγ-immunoreactive dendrites that occupy lamina IIi (Gutierrez-Mecinas et al. 2014).

For the analysis of eGFP expression among retrogradely labelled neurons, we used sections that had been reacted with NeuN and CTb antibodies, and counterstained with DAPI. Because the AAV.flex.eGFP injection did not always occupy the entire mediolateral width of the dorsal horn, we restricted the analysis to the region that contained a high density of eGFP^+^ cells (Gutierrez-Mecinas et al. 2017), and this was initially plotted onto an outline of the superficial dorsal horn. Then, the channels corresponding to NeuN and DAPI were viewed, and all neurons for which the bottom surface of the nucleus lay between the reference and look-up sections were marked on the drawing. The eGFP and CTb channels were then examined, and for each of the selected neurons, the presence or the absence of both eGFP and CTb was determined. A similar analysis was carried out for Pax2 and PKCγ on sections that had been reacted with the other two antibody combinations (NeuN, CTb, Pax2; NeuN, CTb, and PKCγ), except that in this case the disector sample selection involved identifying the bottom surface of the neuronal cell body (immunostained with NeuN), since these sections were not counterstained with DAPI. In addition, the entire mediolateral extent of laminae I–II of the dorsal horn was included in the analysis.

Because we have found that some substance P-expressing neurons in lamina I–II are inhibitory (Gutierrez-Mecinas et al. 2017), we also searched for Pax2 in the nuclei of retrogradely labelled eGFP^+^ cells in scans from the sections that had been reacted for CTb, NeuN, and Pax2. The CTb and eGFP channels were initially viewed with Neurolucida, and 415 lamina I–II CTb^+^/eGFP^+^ cells were identified in the four mice (84–121 cells per mouse). The Pax2 channel was then switched on, and the presence or absence of Pax2 in the nucleus was recorded for each CTb^+^/eGFP^+^ cell.

### Identification of axons belonging to substance P-expressing cells

We found that although intraspinal injection of AAV.flex.eGFP in the Tac1^Cre^ mouse labelled cell bodies that were largely restricted to the injection site (Gutierrez-Mecinas et al. 2017), it also resulted in axonal labelling that extended several segments away from the injection. This presumably results from anterograde transport of the protein within the axons of substance P-expressing neurons. We, therefore, used tissue from the four mice that had only received this type of injection to examine the axonal projections of the eGFP-labelled cells. Since the retrogradely labelled eGFP^+^ cells that were seen in L5 in the first part of the study must have had axons that travelled at least as far as the T13/L1 spinal level, our aim here was to identify axons of eGFP-labelled cells that could have accounted for the uptake and transport of CTb, i.e., those that reached the L1 segment.

For the four mice injected only with AAV.flex.eGFP, we initially determined the extent of the injection site by inspecting non-immunostained sections cut through the L4-5 block and looking for eGFP^+^ cell bodies. We then examined sections that had been reacted with antibodies against eGFP and VGLUT2 from several segments of spinal cord and from the brain, to investigate the distribution of eGFP-labelled axons at different segmental levels rostral and caudal to the injection site. Because eGFP^+^ axons were sparsely distributed in laminae I and II in segments beyond the injection site (see Results), but extended rostrally for several segments in the LSN, we analysed the expression of VGLUT2, substance P and somatostatin in eGFP^+^ axons within the LSN in the L1 segment. This antibody combination was used, because many of the substance P-expressing neurons in the superficial dorsal horn also contain somatostatin (Gutierrez-Mecinas et al. 2017). Co-expression of the peptides in boutons can, therefore, be used to identify axons that are likely to originate from these cells. A single section from the L1 segment of each animal was scanned through a 63× oil-immersion lens (numerical aperture 1.4) to give a *z* series of 45–55 optical sections at 0.3 µm *z* spacing. The eGFP and VGLUT2 channels were initially viewed, and the region corresponding to the LSN was identified based on the distribution of VGLUT2 immunostaining in axon terminals within the nucleus. Approximately 100 eGFP^+^/VGLUT2^+^ boutons were selected throughout the depth of the *z* series. The substance P and somatostatin channels were then viewed, and the presence or absence of each neuropeptide was assessed in all of the selected boutons. Five boutons of each neurochemical type (with or without substance P or somatostatin) from each animal were selected and their *z* depth was determined based on the number of optical sections (0.3 µm separation) in which they appeared. This was carried out to test whether variation in size of the different types of bouton could have resulted in a sampling bias.

### Characterisation of antibodies

The specificity of the CTb antibody is demonstrated by the lack of staining in regions that did not contain injected or transported tracer. The two NeuN antibodies were raised against a recombinant protein consisting of amino acids 1–97 of mouse NeuN protein. When sections are double-stained with either of these antibodies and a well-characterised mouse antibody, each gives a staining pattern that is identical to that seen with the mouse antibody (E Polgár, unpublished observations). The Pax2 antibody is directed against amino acids 188–385 of the mouse protein, and recognizes bands of the appropriate size on Western blots of mouse embryonic kidney (Dressler and Douglass 1992). A Pax2 antibody has recently been shown to label all inhibitory interneurons in the dorsal horn of adult rats (Larsson 2017), and the staining pattern that we saw closely matched that reported by Larsson. The PKCγ antibody was raised against a peptide corresponding to the C terminus of mouse PKCγ, and we have shown that it stains identical structures to those that are labelled with a well-characterised guinea-pig antibody against PKCγ (Sardella et al. 2011). The eGFP antibody was raised against recombinant full-length eGFP and the staining matches that of native eGFP fluorescence. The monoclonal substance P antibody detects the C-terminal 5–8 amino acids of substance P, and does not appear to recognize neurokinin B in tissue sections (McLeod et al. 2000). The somatostatin antibody is reported to show 100% cross-reactivity with somatostatin-28 and somatostatin-25, but none with substance P, and staining is blocked by preincubation with somatostatin (Proudlock et al. 1993). The VGLUT2 antibody was raised against a synthetic peptide from rat VGLUT2, and we have shown that it stains identical structures to a well-characterised rabbit anti-VGLUT2 (Todd et al. 2003). The CGRP antibody is raised against the full-length human α-CGRP and shows 100% cross reactivity with human and rat α-CGRP, human CGRP(8–37), chicken CGRP, and human β-CGRP (manufacturer’s specification). Staining with this antibody closely resembled that seen with a goat anti-CGRP in our previous study (Gutierrez-Mecinas et al. 2014).

### Statistical analysis

Data from the analysis of retrogradely labelled cells were formatted into 2 × 2 contingency tables for each animal, with rows corresponding to the presence or absence of neuronal markers (eGFP, Pax2, and PKCγ) and columns to the presence or absence of CTb immunoreactivity. To determine whether there was a consistent difference in the proportions across the tables for the different cell types, we used the Mantel–Haenszel analysis (McDonald 2014). Breslow–Day testing for homogeneity of the odds ratio was conducted prior to computation of the Mantel–Haenszel odds ratio and 95% confidence intervals.

## Results

### CTb injection sites

**Fig. 1:**
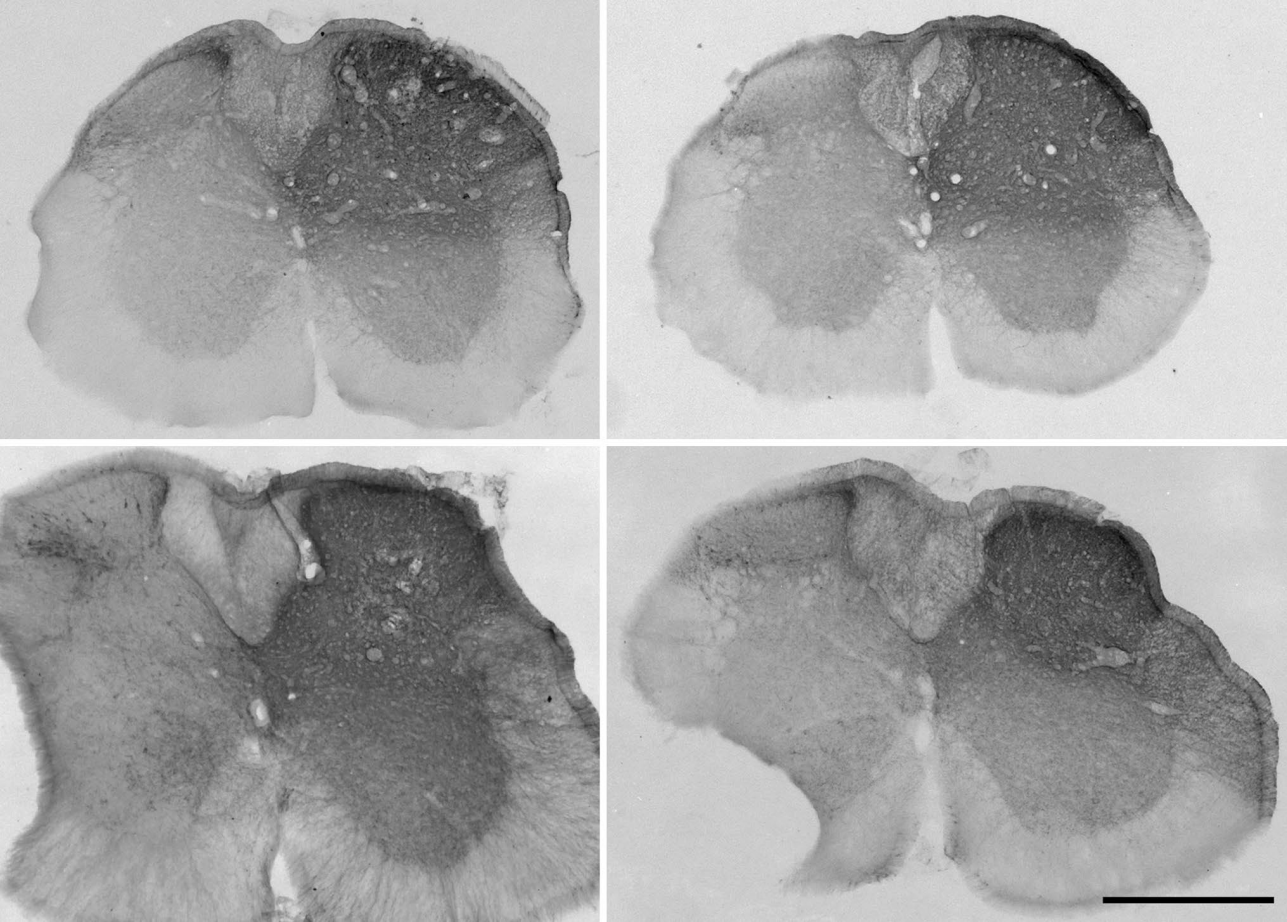
CTb injection sites in the four mice that received injections of CTb and AAV.flex.eGFP. Sections through the T13–L1 block have been reacted by an immunoperoxidase method to reveal CTb. Note that although the extent of the injection site varied between the four mice, it consistently filled the upper part of the dorsal horn (laminae I–IV) and the lateral spinal nucleus on the right side, but did not extend significantly across the midline. Scale bar = 500 µm

The CTb injection sites were found to be located at the T13–L1 segmental levels, based on post mortem identification of the segments defined by dorsal root entry zones, and the characteristic shape of the grey matter in this region (Fig. 1). Although there was some variability in the size of injection sites, as judged from the immunoperoxidase reactions, in all cases, they included the whole of laminae I–V of the right dorsal horn as well as the LSN. In each case, they were restricted to the right side of the spinal cord. We found that the injection site was present on between 4 and 7 of the 60 µm-thick transverse sections obtained from a one in four series, and we, therefore, estimate that the rostrocaudal length of the injection site was between 1000 and 1700 µm.

### Characterisation of retrogradely labelled neurons in the L5 segment

**Fig. 2:**
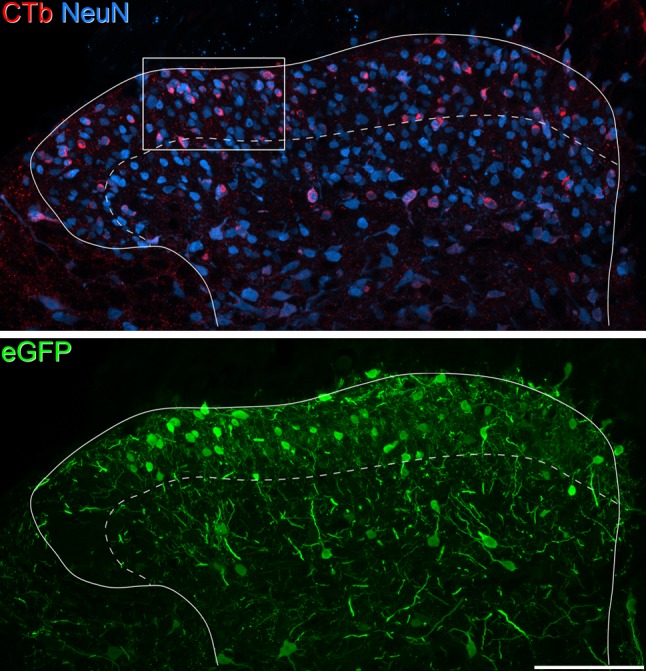
Retrograde labelling with CTb in the L5 segment of a Tac1^Cre^ mouse that had received injections of CTb into T13–L1 and AAV.flex.eGFP into L5. The section has been scanned to reveal CTb (red), NeuN (blue), and eGFP (green). There are numerous CTb-labelled neurons, and the CTb staining appears in the perikaryal cytoplasm of these cells. They are particularly numerous in laminae I–II, occupying the full mediolateral extent of the dorsal horn, and are scattered throughout the deeper laminae. This section is close to the AAV.flex.eGFP injection site, and there are many eGFP^+^ cells, particularly in laminae I and IIo. Note that in this section, the eGFP^+^ cells are not present in the extreme lateral part of the superficial dorsal horn, presumably resulting from lack of spread of the virus from the injection site. The dashed line represents the border between laminae II and III, and the box in the upper image shows the region illustrated in Fig. 3. The image is a projection of 11 optical sections at 1 µm *z* separation, with a pixel size of 0.297 µm. Scale bar = 100 µm

In sections from L5 that had been reacted with each of the antibody combinations, numerous CTb-labelled cells were observed throughout the mediolateral and dorsoventral extent of the superficial dorsal horn, as well as being present in deeper laminae (Fig. 2). Although retrogradely labelled cells were also present on the contralateral side, these were far less numerous, and were largely restricted to the deep dorsal horn and the medial part of the ventral horn. The eGFP injection sites were found to be located within L5, and the distribution of eGFP labelling was very similar to that reported in our previous study (Gutierrez-Mecinas et al. 2017). There was a high density of eGFP^+^ cells in lamina I and the outer half of lamina II (lamina IIo), and scattered cells were present in deeper laminae (Fig. 2). In all cases, these injection sites occupied at least two-thirds of the mediolateral extent of the superficial dorsal horn. The rostrocaudal extent of the eGFP^+^ cells can be estimated, based on the finding that typically, between three and six sections from a one in four series of 60 µm sections contained eGFP-labelled cell bodies in the superficial dorsal horn. This suggests that cells were distributed along a rostrocaudal length of between ~ 700–1400 µm. Since we find that the mean length of the L5 segment is 1.2 mm (EP, unpublished observations), it is likely that the injection site occupies on average approximately one segment in rostrocaudal length.

**Fig. 3:**
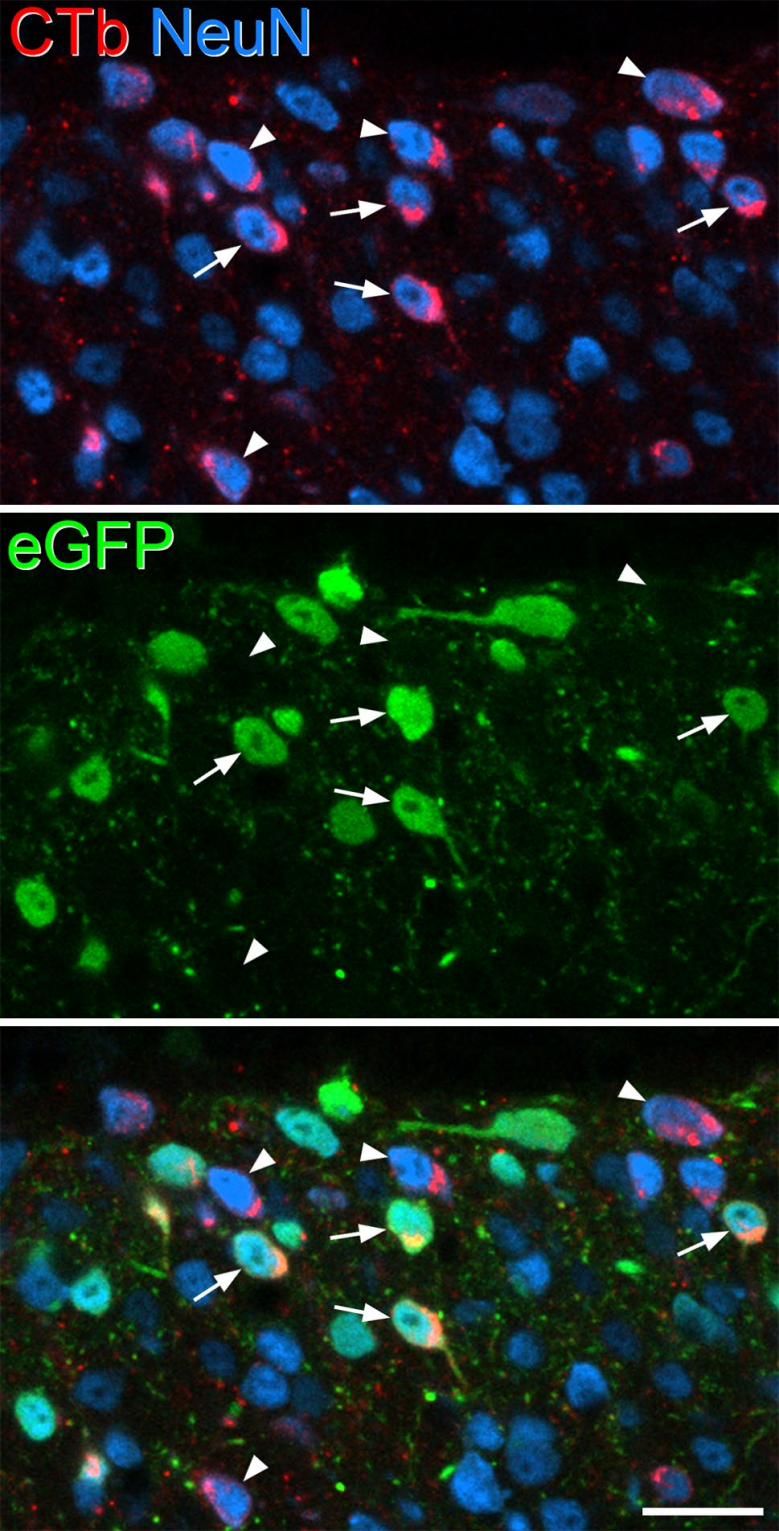
Retrograde labelling of eGFP-expressing cells in a Tac1^Cre^ mouse that had received injections of CTb at T13–L1 and AAV.flex.eGFP at L5. This image shows part of laminae I–II from a single confocal optical section from L5 (pixel size 0.297 µm), and is from the field illustrated in Fig. 2. The section has been immunostained to reveal CTb (red) and NeuN (blue), while eGFP fluorescence appears green. Several CTb-labelled cells are present. Some of those that are also eGFP^+^ are marked with arrows, while some CTb^+^ cells that lack eGFP are marked with arrowheads. Scale bar = 20 µm

**Fig. 4:**
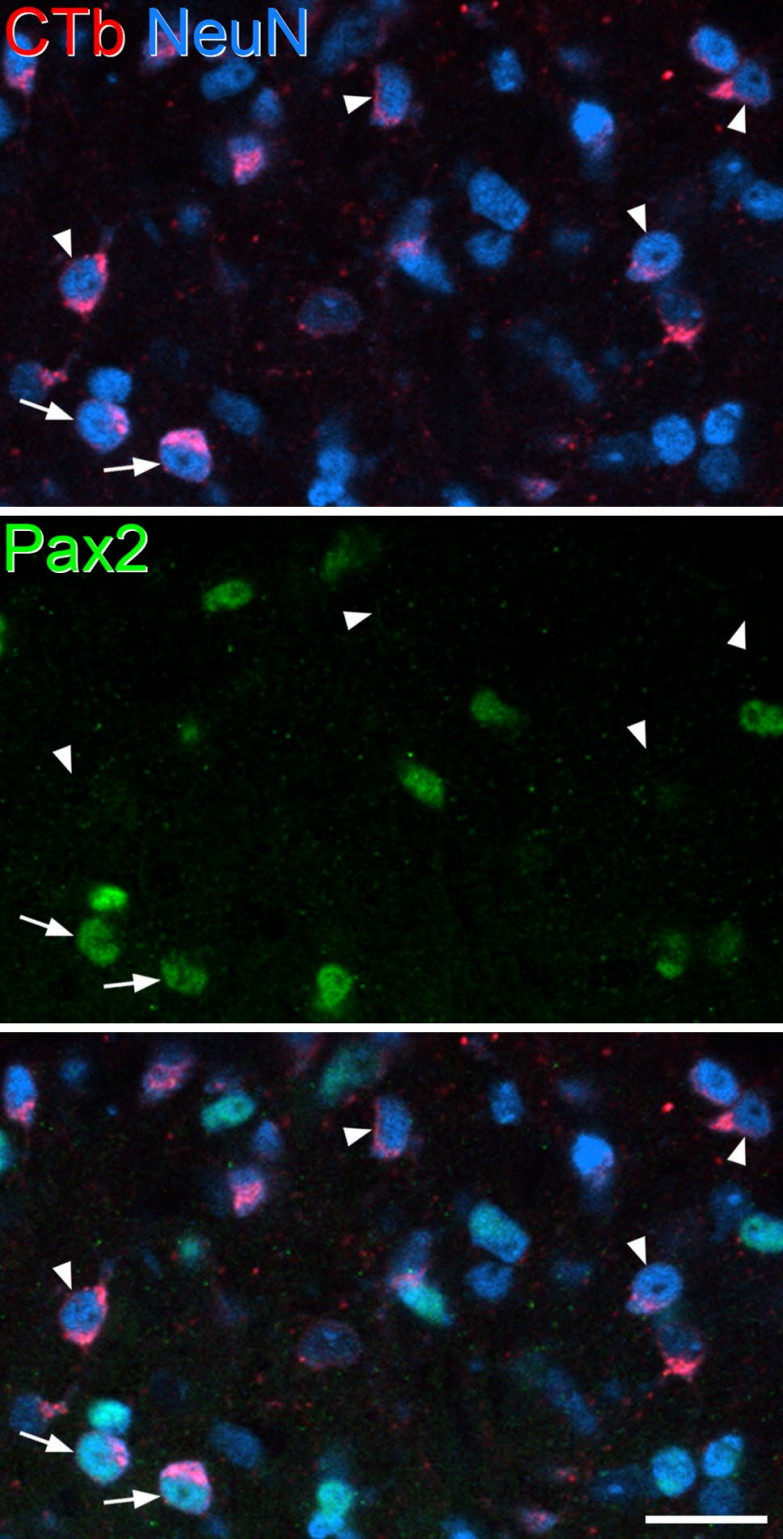
Immunostaining for Pax2 in the L5 segment of a Tac1^Cre^ mouse that had received injections of CTb at T13–L1 and AAV.flex.eGFP at L5. Part of laminae I–II is shown, containing several CTb-labelled neurons. Most of these are Pax2-negative (four of which are indicated with arrowheads), and there are 2 Pax2^+^ CTb-labelled cells (arrows). The image is from a single optical section (pixel size 0.297 µm). Scale bar = 20 µm

**Fig. 5:**
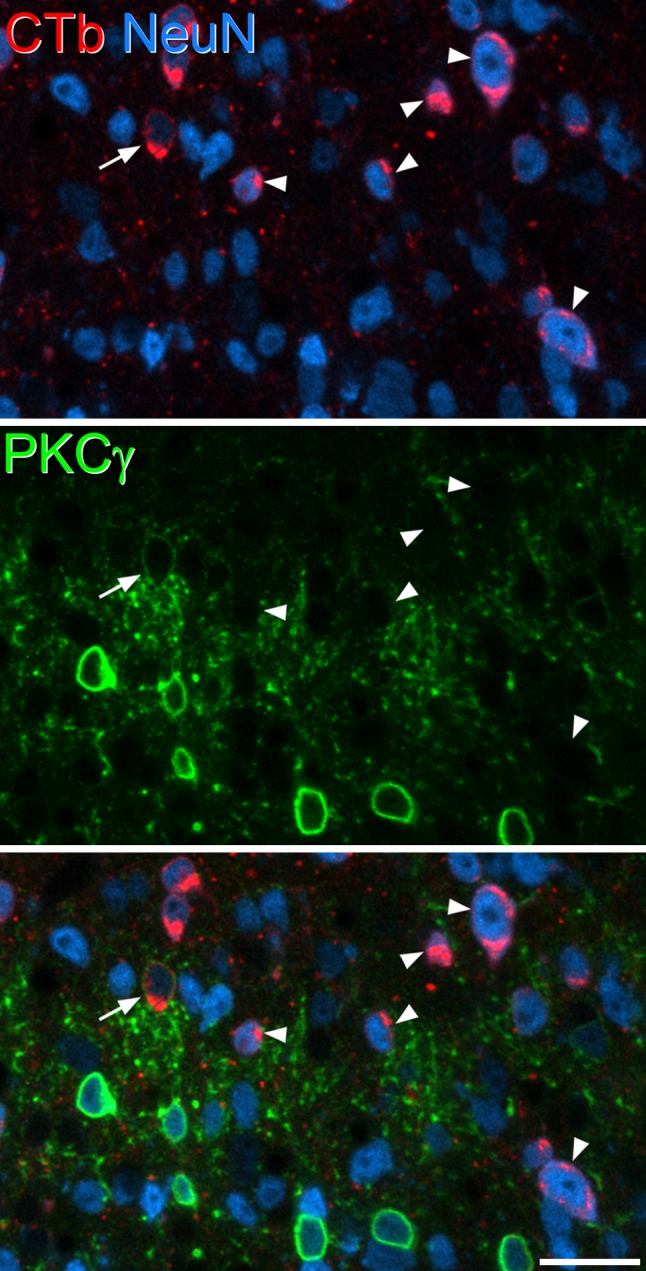
Immunostaining for PKCγ in the L5 segment of a Tac1^Cre^ mouse that had received injections of CTb at T13–L1 and AAV.flex.eGFP at L5. The field shows part of lamina II, and several PKCγ positive cells are visible in the lower half. Most of these are not labelled with CTb, but a single CTb-containing cell with weak PKCγ is present (arrow). Several CTb-labelled cells that lack PKCγ are present, and some of these are marked with arrowheads. The image is from a single optical section (pixel size 0.346 µm). Scale bar = 20 µm

Quantitative data obtained from the analyses of eGFP, Pax2, and PKCγ populations are shown in Tables 2, 3 and 4, and examples of each type of immunostaining are illustrated in Figs. 3, 4, 5. To determine the proportion of all neurons in laminae I–II that were retrogradely labelled with CTb in the four mice, we pooled data from these analyses for each mouse. The mean number of lamina I–II neurons identified in the four mice was 875.8 (range 742–1094), and the proportion of these cells that were CTb immunoreactive was 33.7% (range 30–35.9%). This indicates that around one-third of the neurons in laminae I–II of the L5 segment were retrogradely labelled from an injection into the T13–L1 region, and that despite differences in the extent of the CTb injection between the mice, the percentages of retrogradely labelled neurons were fairly similar. To compare the frequency of retrograde labelling in laminae I and II, we drew the approximate position of the lamina I–II border, which was placed 20 µm below the dorsal surface of the grey matter (Ganley et al. 2015) and analysed cells in these two laminae separately. In lamina I, 44.1% (37.7–48.7%) of neurons were CTb^+^, while for lamina II, the proportion was 29.4% (26.4–31.4%). We also tested whether there was a difference in the proportion of neurons in the medial and lateral halves of the superficial dorsal horn that were CTb^+^ (Petko and Antal 2000), by drawing a line through the middle of the dorsal horn in the dorsoventral axis. The proportions of laminae I–II neurons that were retrogradely labelled were very similar: 36% (31.1–41.5%) for the lateral part and 33.3% (28.6–39.2%) for the medial part.

**Table 2:** CTb and eGFP in Tac1^Cre^ mice injected with CTb and AAV.flex.eGFP

	CTb^+^ neurons	CTb^−^ neurons	Total
eGFP^+^ neurons	26.8 (23–31)	27.5 (17–48)	54.3 (42–76)
eGFP^−^ neurons	59.8 (40–90)	157.5 (120–251)	217.3 (164–341)
Total	86.5 (63–118)	185 (137–299)	271.5 (206–417)

Contingency table showing the mean number of cells containing CTb and/or eGFP that were identified in laminae I–II in the stereological analysis of sections through the L5 segment. The ranges are indicated in brackets. Data were obtained from the four mice that were injected with CTb and AAV.flex.eGFP. Note that the analysis involved the region of the superficial dorsal horn (laminae I–II) that contained a high density of eGFP-labelled cells

**Table 3:** CTb and Pax2 in Tac1^Cre^ mice injected with CTb and AAV.flex.eGFP

	CTb^+^ neurons	CTb^−^ neurons	Total
Pax2^+^ neurons	8.5 (3–12)	57.3 (39–71)	65.8 (49–80)
Pax2^−^ neurons	89 (76–112)	116 (89–144)	205 (178–256)
Total	97.5 (82–121)	173.3 (128–215)	270.8 (227–336)

Contingency table showing the mean number of cells containing CTb and/or Pax2 that were identified in laminae I–II in the stereological analysis of sections through the L5 segment. The ranges are indicated in brackets. Data were obtained from the four mice that were injected with CTb and AAV.flex.eGFP

**Table 4:** CTb and PKCγ in Tac1^Cre^ mice injected with CTb and AAV.flex.eGFP

	CTb^+^ neurons	CTb^−^ neurons	Total
PKCγ^+^ neurons	8 (6–12)	44.3 (33–58)	52.3 (45–65)
PKCγ^−^ neurons	107 (82–142)	176.3 (154–199)	283.3 (260–310)
Total	115 (89–154)	220.5 (187–257)	335.5 (309–375)

Contingency table showing the mean number of cells with CTb and/or PKCγ-immunoreactivity that were identified in laminae I–II in the stereological analysis of sections through the L5 segment. The ranges are indicated in brackets. Data were obtained from the four mice that were injected with CTb and AAV.flex.eGFP

Within the three neuronal populations that we examined, we found substantial differences in the proportions of cells that were retrogradely labelled with CTb. The results for eGFP^+^ cells are shown in Table 2. In the sections used to analyse these cells, 32.2% of all neurons (range 23.7–36.5%) were CTb immunoreactive and 20.3% (18.2–21.7%) were eGFP^+^. The proportion of lamina I–II neurons that were eGFP^+^ is very similar to the value that we previously reported (19.6%) following injection of AAV.flex.eGFP into the Tac1^Cre^ mouse (Gutierrez-Mecinas et al. 2017). However, there was a clear difference in terms of retrograde labelling between those cells with eGFP and those without. Among eGFP^+^ cells, 51.3% (36.8–59.5%) were CTb immunoreactive, while for eGFP-negative cells, the corresponding proportion was 27.5% (24.1–32.8%). These proportions were significantly different (*p* < 0.001; Table 5), showing that eGFP^+^ cells were more likely to be retrogradely labelled when compared to other neurons in laminae I–II (Fig. 3). Cells that were eGFP^+^ accounted for 32.2% (23.7–36.5%) of the CTb-labelled lamina I–II neurons in these sections. None of the 415 CTb^+^/eGFP^+^ cells that were identified in the sections reacted for CTb, NeuN, and Pax2 were Pax2^+^, indicating that all of the retrogradely labelled substance P cells were excitatory neurons.

**Table 5:** Analysis of odds ratios for colocalisation of CTb and different neuronal markers

Neuronal marker	Breslow–Day significance	Mantel–Haenszel test of conditional independence			Estimate of common odds ratio	95% confidence interval
		*χ* ^2^_MH_	*df*	Significance		
eGFP	0.159	36.1	1	< 0.001	2.55	1.88–3.46
Pax2	0.170	78.0	1	< 0.001	0.19	0.13–0.28
PKCγ	0.676	37.2	1	< 0.001	0.30	0.20–0.45

In sections that were used to analyse Pax2, 36.3% (31.5–43.6%) of lamina I–II neurons were CTb-labelled (Table 3). We also found that 24.2% (21.6–27.3%) of all neurons were Pax2-immunoreactive, which is consistent with our previous estimate, based on immunostaining for GABA and glycine, that 26% of the neurons in laminae I–II are inhibitory (Polgár et al. 2013). Among Pax2^+^ cells, 13.3% (4.8–20.4%) were CTb immunoreactive, while the corresponding proportion for Pax2-negative cells was 43.5% (40.1–50%). These proportions were significantly different (*p* < 0.001; Table 5), showing that inhibitory neurons were significantly less likely to be retrogradely labelled when compared to excitatory neurons in laminae I–II (Fig. 4). Pax2-immunoreactive cells accounted for 8.7% (3.7–13.6%) of the CTb-labelled neurons in laminae I–II, indicating that over 90% of the retrogradely labelled cells were excitatory neurons.

In the sections reacted to reveal PKCγ, 34.2% (28.8–45.2%) of lamina I–II neurons were CTb-labelled and 15.5% (13.2–17.3%) were PKCγ-immunoreactive (Table 3). Among the PKCγ cells, 15.9% (10.8–26.7%) were CTb-labelled, and these cells generally showed weak PKCγ-immunoreactivity. For PKCγ-negative cells, the proportion that were CTb-labelled was 37.5% (31.5–48%). Again, these proportions were significantly different (*p* < 0.001; Table 5), showing that PKCγ-expressing neurons were significantly under-represented among those that were retrogradely labelled (Fig. 5). PKCγ cells accounted for 6.9% (5.9–7.9%) of the CTb-labelled neurons in laminae I–II.

### eGFP-labelled axons

**Fig. 6:**
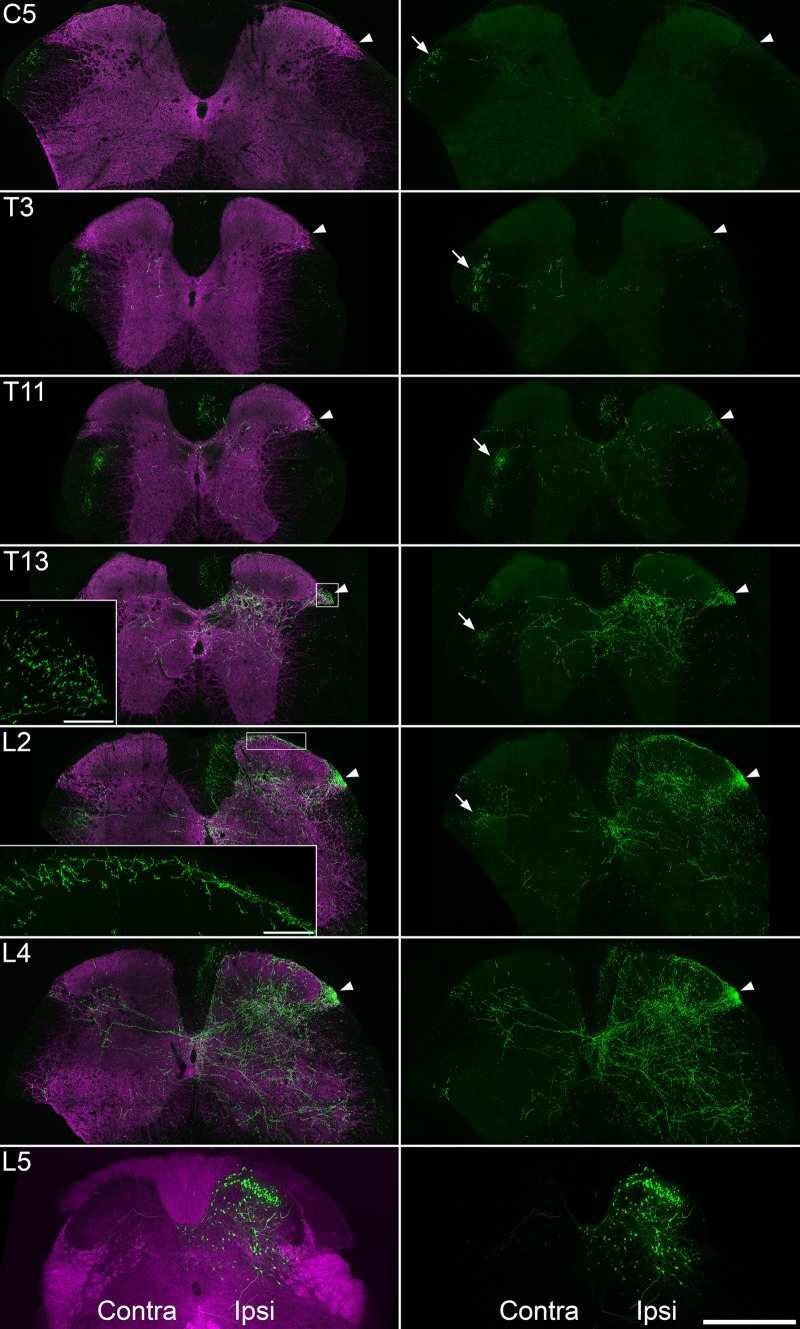
eGFP labelling in the spinal cord of a Tac1^Cre^ mouse that survived for 4 weeks after an intraspinal injection of AAV.flex.eGFP. The pattern of labelling is shown at several levels. For the injection site, which was in the caudal part of the L5 segment, the left panel shows native eGFP fluorescence (green) combined with a dark-field transmitted image (magenta), while the right panel shows eGFP. For all other segments, the left panel shows immunostaining for eGFP (green) and VGLUT2 (magenta), while the right panel shows eGFP immunoreactivity. Close to the injection site in L5, there are numerous eGFP^+^ cells on the ipsilateral (ipsi) side, which are concentrated in the superficial dorsal horn, but scattered in deeper laminae. Axonal eGFP labelling is seen at all of the other segmental levels illustrated. There is a cluster of axons in the dorsal part of the lateral funiculus on the contralateral (contra) side (arrows), which can be clearly seen from L2 to C5, and this presumably consists of ascending spinoparabrachial fibres. There is a dense bundle of axons that ascends within the ipsilateral LSN (arrowhead), and although this progressively diminishes further away from the injection site, it is clearly visible at T13. Individual eGFP-labelled axons can be seen in the ipsilateral LSN in more rostral segments. Scattered axons are also present in the ipsilateral dorsal horn. These are most numerous in L4 and progressively diminish up to T11. Although some of these are in the superficial dorsal horn, these are virtually restricted to lamina I and do not extend beyond T13. There are few eGFP axons in lamina II in L2 and virtually none in this lamina in more rostral segments. Insets show higher magnification views of the axonal labelling in the ipsilateral LSN at the T13 level and in the superficial dorsal horn at L2. Pixel sizes were 0.83 µm (C5, T3, T13), 0.415 µm (T11) 0.208 µm (L2, L4), and 0.503 µm (L5). Scale bars = 500 µm for the main part of the figure and 50 µm for the insets

**Fig. 7:**
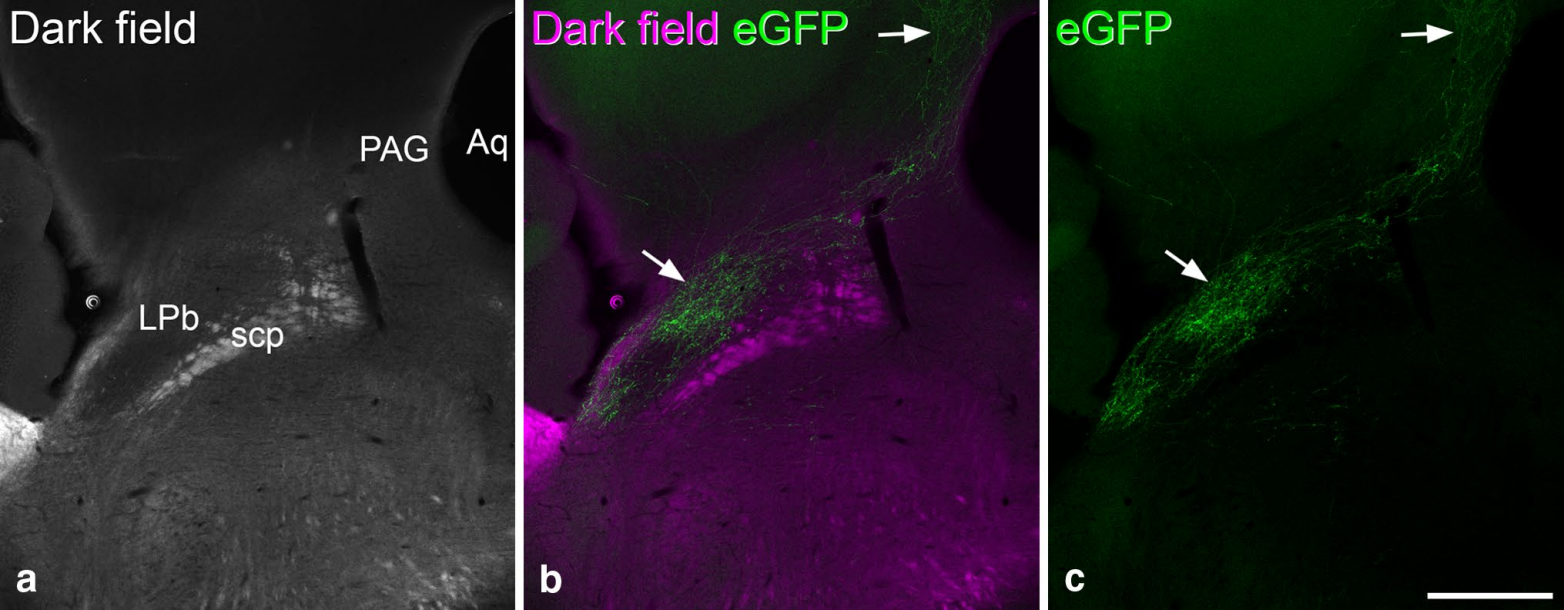
eGFP-labelled axons in the brain of a Tac1^Cre^ mouse that had received an intraspinal injection of AAV.flex.eGFP with a 4-week survival. **a** Dark-field image through the region surrounding the superior cerebellar peduncle (scp) includes the lateral parabrachial area (LPb), the caudal part of the periaqueductal grey matter (PAG) and part of the aqueduct (Aq). **b**,** c** eGFP labelling (green) is shown in a single confocal optical section (pixel size 2.372 µm) with and without the dark-field image (magenta). A dense plexus of labelled axons can be seen in the LPb, and this extends into the PAG (both marked with arrows). These images are taken from the same animal as that illustrated in Fig. 6, which had survived for 29 days after the intraspinal injection. Scale bar = 500 µm

In the four mice that only received injections of AAV.flex.eGFP, injection sites were located in the L5 segment in each case, and were largely restricted to the right dorsal horn, although a few strongly labelled cells were present in the medial dorsal horn on the left side in two mice. In addition to brightly fluorescent cells within the injection site, some labelled cells were seen in the LSN and in the contralateral dorsal horn, and these may have been labelled through retrograde transport from axons entering the injection site. However, eGFP-labelled cell bodies were not seen in any of the other segments examined. In all of the animals, there was extensive axonal staining that could be seen in segments both rostral and caudal to the injection site. The pattern of eGFP labelling in rostral segments in one of the 4-week survival mice is illustrated in Fig. 6. The distribution of eGFP^+^ axons was generally similar between the four mice, but was considerably stronger and more extensive in the two animals that had survived 4 weeks after injection. There was consistently very dense labelling in the ipsilateral LSN in lumbar segments, and labelled axons within the LSN extended at least as far as T13 in the two mice with 1-week survival, and as far as upper thoracic segments in the other two mice (see below). The eGFP labelling within the LSN included both varicosities and intervaricose axons. There were also numerous eGFP^+^ axon branches that ramified in lamina I and these extended for at least three segments rostral to the injection site (i.e., into the L2 segment). Although we did not analyse these formally, we noted that the great majority of the boutons in lamina I were VGLUT2^+^. In addition, there was a diffuse plexus of axons in deeper laminae (III–V) and scattered axons in the intermediate grey matter. However, few eGFP-labelled axons were visible in lamina II at distances more than one segment away from the injection site. In addition, in all animals, there was a distinct and relatively compact bundle of non-varicose axons in the dorsal part of the lateral funiculus on the contralateral (left) side that extended at least as far as T3 in the 1-week survival animals, and as far as C1 in the 4-week survival mice. The location of this bundle closely matches the distribution of antidromic activation sites for contralateral lamina I neurons that were found to project to the midbrain in the rat (McMahon and Wall 1983, 1985). Numerous eGFP-labelled axons were seen in the brains of the two mice that survived 4 weeks after intraspinal injection of AAV.flex.eGFP. Although we did not perform a detailed analysis, we observed many labelled axons with varicosities in regions that are known to receive input from projection neurons in laminae I and III–IV (Todd et al. 2000), including the medullary reticular formation, the LPb and the PAG (Fig. 7). This indicates that some of the eGFP-labelled cells were projection neurons belonging to the spinoparabrachial and spinomesencephalic tracts, and these are likely to have included the large lamina I neurons, together with at least some of those in deeper laminae (III–V). The labelling of projection neurons in the Tac1^Cre^ mouse is consistent with the report that some spinopabrachial neurons express substance P (Blomqvist and Mackerlova 1995; Cameron et al. 2015). No eGFP^+^ axons were seen in the brains of the two mice that survived 1 week after the intraspinal injection, and this indicates that a longer time is required to reveal axons and terminal arborisations within the brain.

**Fig. 8:**
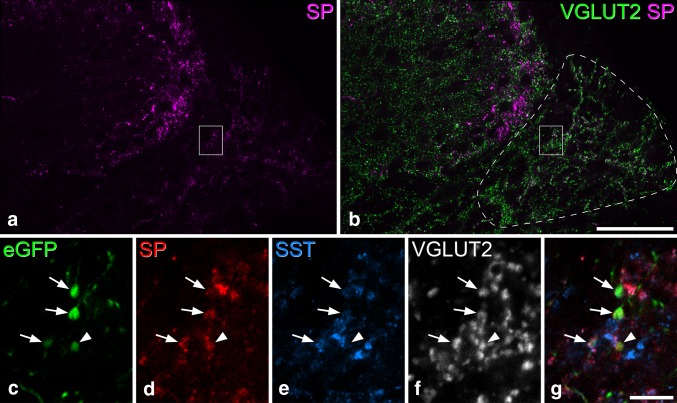
Neuropeptides in eGFP-labelled axons in the LSN. **a**,** b** Confocal image showing the lateral part of the dorsal horn and lateral spinal nucleus (LSN, indicated by the dashed line in **b**) in a section from the L1 segment of a Tac1^Cre^ mouse that had received an intraspinal injection of AAV.flex.eGFP. Immunostaining for substance P (SP, magenta) and eGFP (green) is shown. Note the intense labelling for substance P in profiles in the superficial dorsal horn, and the much weaker staining in the LSN. The box indicates the region of LSN that is shown at higher magnification in **c**–**g**.** c**–**g** These images show immunostaining for eGFP (green), substance P (red), somatostatin (SST, blue), and VGLUT2 (grey). Four eGFP-labelled boutons, all of which are VGLUT2-positive, are indicated. Three of these (shown with arrows) are immunoreactive for both substance P and somatostatin, while one (arrowhead) is only immunoreactive for substance P. All images are from a single confocal optical section (pixel size 0.082 µm). Scale bars **a**,** b** = 50 µm; **c**–**g** = 5 µm

Many eGFP^+^ axons were seen in the LSN ipsilateral to the injection site (Fig. 6). These were most numerous close to the injection site and gradually decreased in density in more rostral and caudal segments. In the caudal direction, eGFP^+^ axons extended at least as far as the S2 segment (data not shown). The eGFP labelling within the LSN consisted of both intervaricose segments and axonal boutons, and the latter could be identified based on the presence of VGLUT2 (Fig. 8). In three of the mice, we estimated the proportion of eGFP^+^ varicosities that were VGLUT2-immunoreactive in the ipsilateral LSN of the L1 segment. We counted between 91 and 115 varicosities per mouse (mean 102) and found that 91.4–95% (mean 93.3%) of these were VGLUT2^+^. Between 86 and 107 (mean 97, *n* = 4 mice), eGFP^+^/VGLUT2^+^ boutons in the ipsilateral LSN at L1 were analysed for neuropeptide expression. Within this population, 72% (69–84%) were substance P immunoreactive, and 75% (57–87%) of these were also somatostatin immunoreactive. Very few of the eGFP^+^ boutons (~ 5%) were somatostatin immunoreactive and substance P negative. The length of boutons along the *z*-axis varied little among the different neurochemical types. Mean values were 1.5 µm for substance P^+^/somatostatin^+^, 1.6 µm for substance P^+^/somatostatin^−^, and 1.5 µm for substance P^−^/somatostatin^−^. Differences in *z*-axis length of boutons is, therefore, unlikely to have resulted in a bias towards any of these populations.

**Fig. 9:**
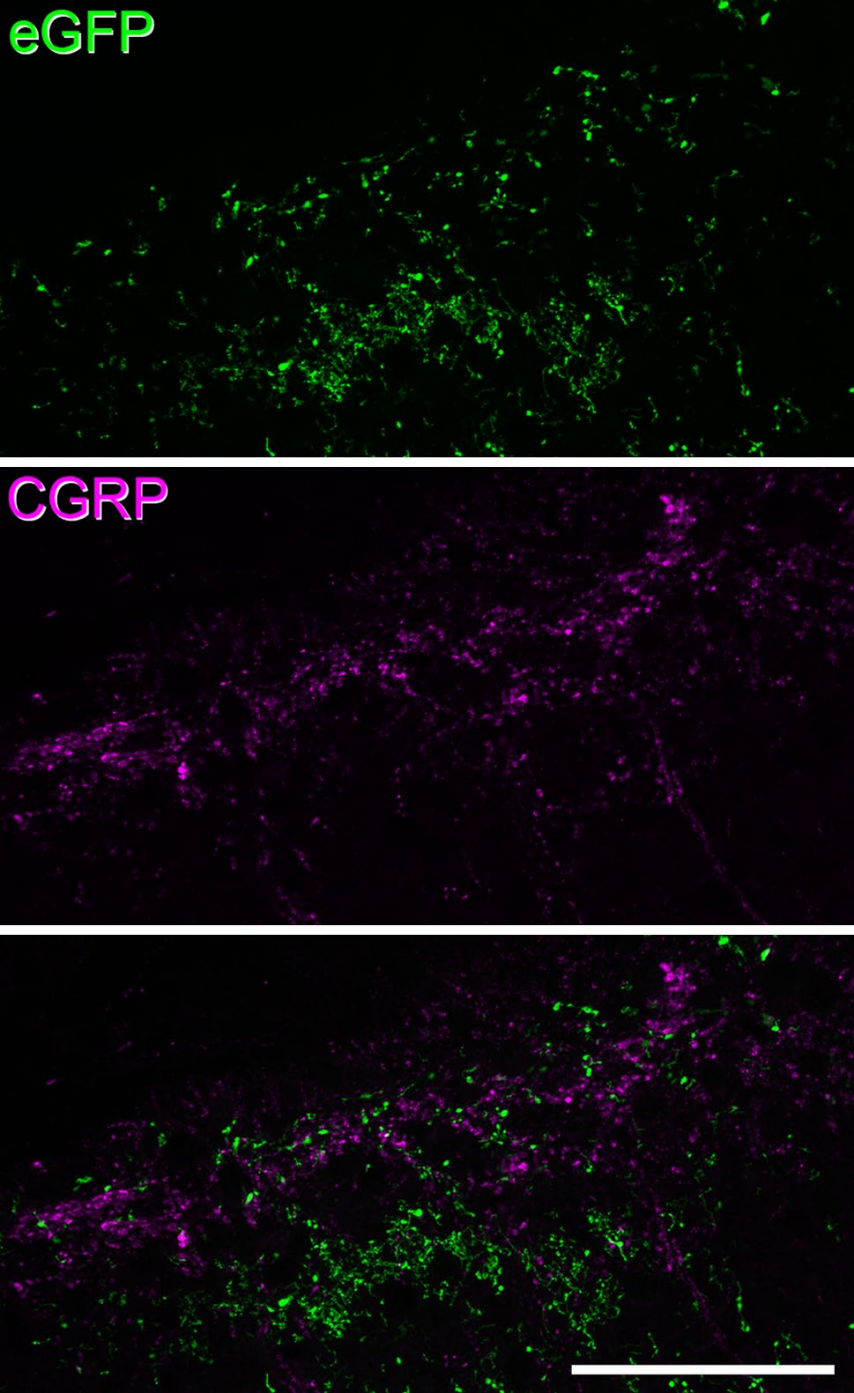
Lack of eGFP labelling in CGRP-containing primary afferents. Confocal images showing immunoreactivity for eGFP (green) and CGRP (magenta) in the superficial dorsal horn from a Tac1^Cre^ mouse that had received an intraspinal injection of AAV.flex.eGFP. The section is taken from the L4-L5 junction, just rostral to the injection site. Note that although there are numerous eGFP- and CGRP-immunoreactive axons, there is no colocalisation of the two markers. The images are projections of five optical sections at 1 µm *z* spacing (pixel size 0.16 µm). Scale bar = 50 µm

We used two approaches to determine whether any of the eGFP-labelled axons were of primary afferent origin. First, we examined the ipsilateral L4 and L5 dorsal root ganglia from two of the injected mice. We did not detect any GFP^+^ cell bodies in any of these ganglia (data not shown). In addition, we analysed sections from the injected spinal cord segments that had been reacted for CGRP, which is coexpressed with substance P in primary afferents (Ju et al. 1987). Although numerous CGRP immunoreactive axons were seen in the superficial dorsal horn, none of these was eGFP^+^ (Fig. 9). This suggests that there was little or no transfection in substance P-expressing primary afferents or their central terminals.

## Discussion

The main findings of this study are: (1) that around 30% of neurons in laminae I–II of the L5 segment were retrogradely labelled following injection of CTb into the ipsilateral side of the spinal cord at the T13–L1 level; (2) that among these neurons, substance P-expressing excitatory neurons were significantly over-represented, whereas inhibitory interneurons and PKCγ-expressing excitatory interneurons were under-represented; (3) that intraspinal injection of AAV.flex.eGFP into the L5 dorsal horn of Tac1^Cre^ mice resulted in sparse axonal labelling in lamina II more than a segment away from the injection site; and (4) that this injection labelled numerous axons in the ipsilateral LSN several segments rostrally, many of which were immunoreactive for both substance P and somatostatin, and, therefore, likely to have originated from substance P-expressing interneurons in the superficial dorsal horn.

### Propriospinal Tac1^+^ neurons and the LSN

In the present study, we used injections of AAV.flex.eGFP into the Tac1^Cre^ mouse to identify the cell bodies of substance P-expressing neurons (Gutierrez-Mecinas et al. 2017). An additional advantage of this approach is that the anterograde transport of eGFP within transfected cells allowed us to follow the axons of substance P-expressing neurons with cell bodies that were located close to the injection site. As reported previously, we found that injection of the viral vector resulted in labelling not only of neurons in laminae I–II, but also of cells in deeper laminae. Although the labelled cells included projection neurons, the great majority of those in the superficial dorsal horn will have been substance P-expressing excitatory interneurons (Gutierrez-Mecinas et al. 2017). Substance P-expressing primary afferents do not enter the LSN (Bresnahan et al. 1984; Cliffer et al. 1988; Giesler and Elde 1985; Sikandar et al. 2017), and we did not detect CGRP in any of the eGFP-labelled boutons, indicating that these did not originate from primary afferents. The eGFP-labelled axons within LSN must, therefore, have originated from spinal neurons. Some of these axons may have been collaterals of the spinoparabrachial neurons (Szucs et al. 2010), or have arisen from neurons in deeper laminae or from the LSN itself. However, it is likely that many were derived from interneurons in laminae I–II, since 75% of the substance P-immunoreactive axonal boutons that we identified also contained somatostatin. The rationale for using this approach is that somatostatin expression in the spinal cord is largely restricted to laminae I–II, where it can be detected in at least 70% of the excitatory substance P-containing neurons (Gutierrez-Mecinas et al. 2017). Importantly, somatostatin does not co-localise with substance P in primary afferents (Li et al. 2016; Usoskin et al. 2015; Hokfelt et al. 1976; Nagy and Hunt 1982), and is thought not to be expressed in lamina I projection neurons (Duan et al. 2014) or LSN neurons (Allen Spinal Cord Atlas. http://mousespinal.brain-map.org/). Although there are scattered somatostatin cells in deeper dorsal horn laminae, most of these are inhibitory (Duan et al. 2014; Proudlock et al. 1993). While we cannot rule out a contribution from cells in deeper laminae, it is most likely that boutons in the LSN that contained VGLUT2 together with both these neuropeptides originated from substance P-expressing excitatory interneurons in laminae I–II. Although it might be expected that all axonal boutons belonging to Tac1^+^ cells would contain substance P, we have found that the level of immunoreactivity for the peptide is generally much lower in axons belonging to substance P-expressing interneurons than in primary afferents (MGM and AJT, unpublished observations), and it was presumably below the detection threshold for immunocytochemistry in some boutons belonging to these cells.

Szucs et al. (2010) have reported that axon collaterals of some lamina I projection neurons arborise within the dorsal horn, and it is likely that both these and substance P-expressing interneurons contributed to the axonal staining seen in this region close to the injection site. However, we observed relatively few eGFP-labelled axons within the superficial dorsal horn in rostral lumbar segments, and these were largely restricted to lamina I. Taken together with our finding of extensive axonal labelling in the LSN, this strongly suggests that the LSN represents a major route through which substance P-expressing cells in laminae I–II of the L5 segment were retrogradely labelled following injection of CTb into the T13/L1 region and that this nucleus is an important target for propriospinal substance P-expressing excitatory interneurons.

The discrepancy between the results reported by Bice and Beal (1997b) and those of Petko and Antal (2000, 2012) are likely to be explained by our finding that at least some of the propriospinal neurons (those that express substance P) give rise to axons that arborise within the LSN, but do not re-enter the superficial dorsal horn in significant numbers. Although substance P-expressing cells only accounted for around one-third of the propriospinal neurons in laminae I–II, if the remaining propriospinal neurons have intersegmental projections that are restricted to the LSN, this would explain the lack of retrogradely labelled cells more than a segment away from tracer injections that are limited to the dorsal horn (Petko and Antal 2000, 2012).

We found that around 34% of neurons in laminae I–II of the mouse L5 segment project at least 5 segments rostrally, and this is considerably higher than the proportion (~ 12%) of superficial dorsal horn neurons in the L1 segment that were labelled from T5 in the rat, a distance of nine segments (Bice and Beal 1997b). Although there could be differences related to the species and the segment into which the injection was made, the most likely explanation for the higher proportion that we see is that there is a gradual reduction in the number of cells projecting to more distant segments. This would be consistent with our finding that eGFP labelling in the LSN progressively decreases with increasing distance from the injection site.

### Role of the LSN

The LSN, which was originally identified by Gwyn and Waldron (1968), has been the subject of several anatomical and functional studies. It is present throughout the rostrocaudal length of the spinal cord in rodents, but is displaced by the lateral cervical nucleus in the upper cervical cord (Giesler and Elde 1985). Anatomical studies have shown that it receives very little direct primary afferent input (Bresnahan et al. 1984; Sikandar et al. 2017; Cliffer et al. 1988), with the exception of a few visceral afferents (Neuhuber 1982; Sugiura et al. 1989). However, it has a rich network of neuropeptide-expressing axons that are thought to originate from local dorsal horn neurons (Cliffer et al. 1988; Giesler and Elde 1985; Olave and Maxwell 2004). Many LSN neurons project supraspinally to the brainstem and diencephalon (Burstein et al. 1990; Menetrey et al. 1980, 1982; Olave and Maxwell 2004). Interestingly, many of these cells (including the projection neurons) are immunoreactive for the neurokinin 1 receptor (Littlewood et al. 1995; Olave and Maxwell 2004; Ding et al. 1995), and application of substance P depolarises cells in this nucleus (Jiang et al. 1999). Since substance P-expressing primary afferents do not enter the LSN, the main source of substance P to activate the receptor is presumably spinal interneurons, including those with long propriospinal axons.

There have been relatively few in vivo electrophysiological studies of LSN neurons. Menetrey et al. (1980) recorded from 15 spinoreticular neurons in the midlumbar part of the rat LSN and found that only 2 of these had clear receptive fields, which covered the whole ipsilateral hindlimb. The remaining units had poorly defined fields on the proximal part of the limb that could extend onto the scrotum or base of the tail. These responded variably to stimuli applied to the skin and deep tissues, including extreme joint movement. Sikandar et al. (2017) recently reported that LSN neurons in the rat were activated mainly by noxious stimulation of deep tissues. However, they could be sensitised by intramuscular hypertonic saline, following which they developed large cutaneous receptive fields, and responded to brushing of the skin, as well as showing heightened responses to noxious stimuli.

Our results suggest that many substance P-expressing cells send ascending and descending axons into the LSN and that these give rise to boutons (and presumably synapses) over a considerable rostrocaudal extent of the nucleus. As discussed above, a similar arrangement may also apply to other propriospinal neurons in laminae I–II. The propriospinal input to LSN is likely to be predominantly excitatory, since excitatory neurons outnumbered inhibitory neurons among those with long propriospinal connections. However, our finding that 13% of inhibitory neurons have long propriospinal projections suggests that these may give rise to large inhibitory receptive fields for LSN cells. Until now, it has been assumed that the input to LSN from superficial dorsal horn neurons was exclusively from cells in the same or nearby segments, because Cliffer et al. (1988) saw no reduction in neuropeptide content in the LSN at L4 following spinal lesions (including complete transection) at low thoracic or upper lumbar levels. Our findings show that there are significant propriospinal inputs from substance P-expressing neurons that can extend for at least five segments. However, these are presumably greatly outnumbered by inputs arising from the same or adjacent segments, and this would explain the lack of neuropeptide depletion seen by Cliffer et al. Since substance P-expressing interneurons in laminae I–II can respond to a variety of noxious stimuli delivered to the skin (Gutierrez-Mecinas et al. 2017), these long propriospinal connections are likely to contribute to the extensive (whole limb) receptive fields seen for some LSN cells by Menetrey et al. (1980) and the development of large receptive fields following sensitisation by intramuscular hypertonic saline, reported by Sikandar et al. (2017). Although PKCγ-expressing neurons were under-represented among the cells with long propriospinal projections, ~ 15% of these cells were retrogradely labelled. PKCγ neurons are thought to be activated by innocuous tactile stimuli (Neumann et al. 2008), and if the propriospinal PKCγ cells innervate the LSN, they may give rise to the large brush-sensitive receptive fields that are seen following sensitisation (Sikandar et al. 2017).
